# Dopamine D1-D2 receptor heteromer signaling pathway in the brain: emerging physiological relevance

**DOI:** 10.1186/1756-6606-4-26

**Published:** 2011-06-13

**Authors:** Ahmed Hasbi, Brian F O'Dowd, Susan R George

**Affiliations:** 1 Centre for Addiction and Mental Health, Toronto, ON M5T 1R8, Canada; 2 Department of Pharmacology and Toxicology, University of Toronto, Toronto, ON M5S 1A8, Canada; 3 Department of Medicine, University of Toronto, Toronto, ON M5S 1A8, Canada

## Abstract

Dopamine is an important catecholamine neurotransmitter modulating many physiological functions, and is linked to psychopathology of many diseases such as schizophrenia and drug addiction. Dopamine D1 and D2 receptors are the most abundant dopaminergic receptors in the striatum, and although a clear segregation between the pathways expressing these two receptors has been reported in certain subregions, the presence of D1-D2 receptor heteromers within a unique subset of neurons, forming a novel signaling transducing functional entity has been shown. Recently, significant progress has been made in elucidating the signaling pathways activated by the D1-D2 receptor heteromer and their potential physiological relevance.

## Background

Dopamine plays a key role in the regulation of various physiological functions of normal brain including reward, locomotion, behavior, learning, and emotion. It is not then surprising that the dysregulation of the dopaminergic system has been linked to pathophysiology of many diseases, such as Alzheimer's disease, schizophrenia, Parkinson's disease, attention deficit hyperactivity disorder, depression and drug addiction [1-3], leading to the clinical use of drugs that target dopamine neurotransmission in the treatment of these disorders.

Five subtypes of dopamine receptors (D1R-D5R), belonging to the G-protein-coupled receptor (GPCR) superfamily have been cloned, through which dopamine transduces its various effects. Dopamine receptors are subdivided into D1-like (D1, D5) and D2-like (D2, D3, D4) receptor subclasses [1-3], with the D1 and D2 receptors being the major subtypes. The most studied dopamine signaling pathway is the modulation of cyclic AMP production, with D1-like receptors activating cyclic AMP production through Gs/olf, and D2-like receptors inhibiting adenylyl cyclase (AC) activity through Gi/o proteins [2]. This results in a bidirectional modulation of this pathway and related proteins, such as protein kinase A (PKA) and DARPP-32 (dopamine and cAMP regulated protein) [4]. Other important dopamine signaling pathways have also been reported, including the modulation of the Akt-GSK3 pathway [5] and the activation of the PAR4 signaling pathway [6].

For some actions of dopamine, such as the control of motor behavior [7] or dopamine-mediated reward processes in nucleus accumbens [8], a concomitant stimulation of D1 and D2 receptors is required, a phenomenon known as the "requisite" D1/D2 synergism [9]. In this type of synergism, D1 and D2 receptor-specific drugs potentiate the effect exerted by each other when delivered together, but are ineffective when administered separately [9]. The combined, but not separate, administration of a selective D1 and a selective D2 agonist was shown to be necessary for the dopamine-stimulated expression of immediate-early gene c-fos in striatal neurons [10] and in electro-physiological studies where both receptors were indeed responsible for GABA release in striatum [11]. The participation of both D1 and D2 receptors was also required for evoking neural and behavioral sensitization to cocaine [12] and for evoking the changes in behavior and basal ganglia output [13,14]. All these observations are other evidence for the presence of not only a synergism between dopamine D1 and D2 receptors, but an obligatory participation of both receptors to generate this synergism.

One explanation for how the well documented synergistic effects seen between D1 and D2 receptors [15,16] may be achieved is through the formation of heterooligomers between the two receptors, as it has been shown for many GPCRs [17-19]. Dopamine receptors, all subtypes included, in addition to their ability to exist as homomers, were shown to form different heteromeric complexes with other receptors (reviewed in 20). The presence of D1-D2 receptor heteromers with unique functional properties was first shown in transfected cells using different methods [21-24] as described below. Initially, the notion of heteromerization observed for many GPCRs and its functional relevance was not completely clear in physiological conditions and was in some cases regarded with a degree of skepticism, but at least for the D1-D2 receptor heteromer we have shown evidence of occurrence under physiological conditions in native tissues with emerging important functional relevance.

For D1 and D2 receptors, the presence of two anatomically segregated sets of neurons, forming the striatonigral D1-enriched direct pathway and the striatopallidal D2-enriched indirect pathway is commonly recognized, with D1R localizing to the dynorphin (DYN)-expressing neurons, and D2R localizing to the enkephalin (ENK)-expressing neurons [25,26]. Recent studies emanating from fluorophore-tagged promoter elements of D1R and D2R in bacterial artificial chromosome (BAC) transgenic mice [27] allowed an evaluation of the proportions of striatal neurons expressing D1R, D2R, or both [28-32]. There were, however, variations in the levels of expression of EGFP between one line and another [32], resulting in incomplete labeling of a significant proportion of striatal medium spiny neurons (MSNs) [28]. While this method supported the segregation between the D1-enriched direct pathway and the striatopallidal D2-enriched indirect pathway, a certain fraction of MSNs (~17%) expressing both receptors was predicted in the NAc shell, whereas only ~5-6% of MSNs were calculated to co-express both receptors in the dorsal striatum [30-32]. These BAC-calculated colocalization data are consistent with our data and the numerous other reports indicating a colocalization of D1R and D2R in neurons in culture or in situ with higher D1R and D2R co-localization observed in cultured striatal neurons (60 to 100%) than in the adult striatum [33-40].

### Presence of dopamine D1-D2 receptor heteromers in brain

Several reports indicated the presence of a D1-like receptor activating IP3 production and/or increasing intracellular calcium in neurons in culture or slices from different brain regions, including striatum, hippocampus, and cortex [41-44]. However, the cloned D1R was devoid of such effects when expressed in different host cells (reviewed in 17 and 20) and persisted in a D1 receptor null mouse model [45]. We then demonstrated that dopamine D1 and D2 receptors form functional heterooligomeric complexes in cells and in vivo [21-23,40,46] and that the mobilization of intracellular calcium was in fact a unique signaling pathway resulting from the activation of this D1-D2 heteromeric receptor complex [21,23,40].

The presence of the D1-D2 receptor heteromer was demonstrated by different techniques including coimmunoprecipitating both receptors from rat striatum, as well as from cells coexpressing D1R and D2R [21,40], and by different methodologies using the fluorescence resonance energy transfer (FRET) technique in cells [22,24], in striatal neurons [40,47] and different brain regions [40,46].

Interestingly, in adult rat brain, coexpressed dopamine D1 and D2 receptors were present in a unique subset of neurons coexpressing both DYN and ENK neuropeptides in different brain regions, including nucleus accumbens (NAc), caudate-putamen (CP), ventral pallidum, globus pallidus (GP), and entopeduncular nucleus [46], with some inter-regional variation. The lowest proportion (~6-7%) of D1R-expressing neurons that coexpress D2R was shown in the CP [40,46], whereas the highest proportion (~59%) of D1R-expressing neurons that coexpress D2R was observed in GP [46]. A substantial number (~20-30%) of D1R neurons that coexpress D2R was also observed in NAc [40,46], consistent with the anatomical findings resulting from BAC transgenic mice [30-32].

**Figure 1:**
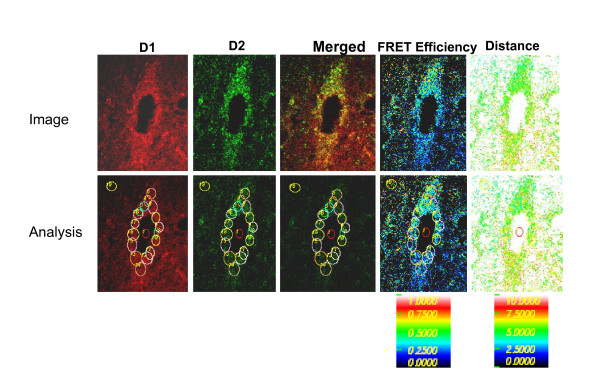
**Example of Confocal FRET analysis of D1 and D2 receptor interaction in a medium spiney neuron from the core region of rat nucleus accumbens**. Anti-D2-Alexa 350 (green) and anti-D1-Alexa 488 (red) were used as donor and acceptor dipoles. The FRET signal was detected and measured in microdomains [regions of interest (ROIs)] within the neuron coexpressing D1 and D2 receptors. Analysis shows the FRET efficiency and the distance separating the dipoles.

The direct interaction of D1R and D2R to form heteromers in brain was shown by confocal FRET technique using two methodologies [40,46,47]. The confocal FRET technique demonstrated clearly and directly the presence of the D1-D2 receptor heteromer in striatal neurons [40,47] and in brain *in situ *[40,46]. In NAc, acceptor photobleaching-based FRET showed a high FRET efficiency of ~21% [46], in the same range (~20%) as with a second quantitative confocal FRET, that further quantified the parameters of the interaction between D1R and D2R to calculate the FRET efficiency and the assessment of the distance separating both fluophore-tagged receptors [40,46]. In NAc, interactions between colocalized D1R and D2R (Figure 1) displayed high FRET efficiency (~20%) and a relative distance of 5-7 nm (50-70 Å) (Table 1), synonymous with a close proximity between D1 and D2 receptors and indicative of D1-D2 heteromer formation. In contrast, although an indication of D1-D2 heteromer formation in CP was observed, the parameters, FRET efficiency (~5%) and the relative distance of 8-9 nm (80-90 Å) between the receptors suggested that in CP either D1R-D2R interaction was weaker, or fewer D1-D2 receptor heteromers were formed, and/or lower order of D1-D2 oligomers than in the NAc was present [40,46].

**Table 1:** Confocal FRET analysis of D1 and D2 receptor interaction

ROI	Donor of FRET	Acceptor of FRET	PFRET	FRET Efficiency	Distance between donor and acceptor (nm)
(1) Donor alone	13.944	0	0	0	10

2	842.685	562.542	529.703	0.357	5.91

3	804.879	488.573	474.042	0.351	5.9

4	830.377	569.241	535.203	0.353	5.924

5	720.099	436.039	410.781	0.319	6.269

6	898.475	482.132	444.885	0.311	6.171

7	964.916	460.029	407.186	0.247	6.875

8	1116.854	399.85	384.365	0.234	6.632

9	951.224	324.177	314.284	0.206	7.145

10	1076.73	341.095	326.925	0.2	7.153

11	976.861	227.299	216.367	0.149	7.789

12	1201.314	363.612	336.45	0.191	7.121

13	998.373	283.121	269.621	0.187	7.197

14	1017.225	303.213	287.876	0.2	6.987

15	816.347	166.339	156.562	0.129	8.069

16	806.034	265.133	251.731	0.19	7.393

17	815.063	349.81	338.709	0.252	6.792

18	833.344	485.752	382.262	0.257	6.946

(19)Non-Specific	95.52	83.573	35.284	0.086	9.168

**Average**	921.8117	382.821	356.88	**0.243117**	**6.83958**

**SEM**	33.82434	29.9949	27.1577	0.018620	0.165392

Confocal FRET analysis of figure 1 shows the relative expression of the donor (D2-Alexa 350, green) and acceptor (D1-Alexa 488, red). The analysis also shows the processed FRET (pFRET), the FRET efficiency and the distances separating the two fluorophore-tagged receptors in each microdomain (ROI), with averages and SEM in the bottom of the table. A distance ~10 nm or higher indicates no FRET.

### D1-D2 receptor heteromer-induced signaling pathway and its physiologic relevance

The specific activation of the D1-D2 receptor heteromer in postnatal striatal neurons [40], and from cells co-expressing D1R and D2R [21,23] resulted in the intracellular release of calcium from stores sensitive to activation of inositol triphosphate receptors (IP3-R). This rise in intracellular calcium was rapid, transient, independent of extracellular calcium influx, and involved the activation of Gq protein, and phospholipase C (PLC) [21,23,40]. This calcium signal resulted in an increase in the phosphorylated-activated form of CaMKIIα in postnatal striatal neurons [40] and rat striatum [23]. The use of dopamine D1^-/-^, D2^-/- ^and D5^-/- ^receptor null mice indicated clearly that the calcium-CaMKIIα signaling pathway exclusively involved both D1R and D2R within a functional complex [23,40], and was different from the calcium signal generated by the activation of D5R or the D2-D5 receptor heteromer [48,49].

Intracellular calcium plays key roles in many neuronal functions including the regulation of synaptic transmission [50]. The intracellular calcium signaling pathway activated through the dopamine D1-D2 receptor heteromer resulted in CaMKIIα activation and BDNF production in striatal neurons in culture as well as in the nucleus accumbens of adult rats, leading ultimately in cultured postnatal striatal neurons to enhanced dendritic branching [40]. Both CaMKIIα and BDNF have been shown to be involved in synaptic plasticity. While evidence has indicated that CaMKIIα is a critical regulator of synaptic plasticity in neurons [51-54] with 50% of CaMKIIα-deficient mice presenting changes in behavior and learning [55], BDNF has been shown to modulate the branching and growth of axons, dendrites and spines (reviewed in 56). For example, BDNF was shown to be released from cell bodies and dendrites of cortical neurons and regulated the branching of dendrites in adjacent neurons [57]. The BDNF effect on the dendritic morphology and also on spine morphology (reviewed in 56) would be of great importance in the modulation of neuronal and synaptic function and plasticity [58]. The neurotrophin signaling transduced through BDNF receptor TrkB has been recently reported to be involved in the control of the size of the striatum by modulating the number of medium spiny neurons (MSNs), with deletion of the gene for the TrkB receptor in striatal progenitors leading to the loss of almost 50% of MSNs without affecting striatal interneurons [59]. Also, the BDNF signaling through TrkB was shown to be involved in the induction and the maintenance of synaptic plasticity, through its long-term potentiation (LTP) component [60]. The other component, long-term depression (LTD) was shown to involve BDNF signaling through the receptor p75 in hippocampal slices from p75-deficient mice [61]. BDNF plays also an important role in the modulation of neurotransmitter release, a key step in synaptic plasticity [56]. The release of glutamate for example involves PLC and BDNF through a mechanism involving a rise in intracellular calcium via a release from IP3 receptor-sensitive stores [62,63]. It is very interesting to draw the parallel between these mechanisms by which CaMKII and BDNF modulate synaptic plasticity and the signaling pathway revealed with the activation of dopamine D1-D2 receptor heteromer in the striatum [40], which also involves PLC, the intracellular calcium release from IP3 receptor-sensitive stores, CaMKII activation and BDNF production. This suggests that the D1-D2 receptor heteromer-mediated signaling pathway may play an essential role in synaptic plasticity, notably in its LTP component [20,40,49], the dysregulation of which may lead to alterations in cognition, learning, and memory that contribute to the pathophysiology of dopamine-related disorders such as schizophrenia or drug addiction [20,40,46,49].

Further, we showed that in rat striatum amphetamine administration significantly increased the affinity of SKF 83959, a specific D1-D2 receptor heteromer agonist [64], by 10-fold for the D1-D2 receptor heteromer and increased the proportion of the D1-D2 heteromer in the agonist-detected high affinity state [46]. GTPγS binding studies indicated that the D1-D2 heteromer was functionally supersensitive in response to repeated increases in dopamine transmission following amphetamine administration [46]. In addition to increasing the activity and sensitivity of D1-D2 receptor heteromers, amphetamine also increased the D1-D2 receptor heteromer density in the NAc as assessed by FRET technique [46].

Interestingly, the increase in the proportion of D1-D2 heteromers in the high affinity state was also detected in schizophrenia globus pallidus (GP) [46]. Amphetamine treatment leading to increased dopamine transmission and behavioral sensitization has been used as an animal model for schizophrenia [65], since schizophrenia has been linked to increased dopamine transmission [66]. Moreover, the different components of calcium signaling, including Gq proteins, PLC, and CaMKII were shown to be affected in the brains of schizophrenia patients [67]. Given these facts, the findings showing an increase in the proportion of D1-D2 heteromers in high affinity state in both schizophrenia and chronic amphetamine treatment may indicate a preponderant role of the D1-D2 receptor heteromer-mediated calcium-CaMKII-BDNF signaling pathway in both drug addiction and schizophrenia.

This D1-D2 receptor heteromer-calcium signal may represent a first common biochemical bridge between the dopaminergic system-CaMKII-BDNF, synaptic plasticity and the occurrence of drug addiction and schizophrenia. The finding that the activation of CaMKIIα was necessary for the induction of behavioral sensitization to drugs [68], a physiological phenomenon that also requires the coactivation of D1 and D2 dopamine receptors [14], provides additional evidence of the important role of dopamine D1-D2 receptor heteromer-calcium signal in drug addiction.

After years of some skepticism surrounding the physiological presence and relevance of GPCR homo- and hetero-oligomers, there is ample evidence for the presence in the brain of a unique entity, the D1-D2 receptor heteromer, with a unique signaling pathway different from the signals generated by each receptor homomer, with a physiological relevance and high importance in at least two major pathologies, schizophrenia and drug addiction, making the D1-D2 receptor an interesting therapeutic target for these disorders.

## Competing interests

The authors declare that they have no competing interests.

## Authors' contributions

All authors read and approved the final manuscript
